# Phosphorus Acquisition Efficiency and Transcriptomic Changes in Maize Plants Treated with Two Lignohumates

**DOI:** 10.3390/plants12183291

**Published:** 2023-09-17

**Authors:** Veronica Santoro, Maria Cristina Della Lucia, Ornella Francioso, Piergiorgio Stevanato, Giovanni Bertoldo, Matteo Borella, Erika Ferrari, Claudio Zaccone, Michela Schiavon, Diego Pizzeghello, Serenella Nardi

**Affiliations:** 1 Dipartimento di Scienze Agrarie, Forestali e Alimentari, University of Torino, Largo Paolo Braccini 2, 10095 Grugliasco, Italy; veronica.santoro@unito.it; 2 Dipartimento di Agronomia, Animali, Alimenti, Risorse Naturali e Ambiente, University of Padova, Viale dell’Università 16, 35020 Legnaro, Italy; mariacristina.dellalucia@phd.unipd.it (M.C.D.L.); stevanato@unipd.it (P.S.); giovanni.bertoldo@phd.unipd.it (G.B.); matteo.borella.1@phd.unipd.it (M.B.); diego.pizzeghello@unipd.it (D.P.); serenella.nardi@unipd.it (S.N.); 3 Dipartimento di Scienze e Tecnologie Agro-Alimentari, University of Bologna, Viale Fanin 40, 40127 Bologna, Italy; ornella.francioso@unibo.it; 4 Dipartimento di Scienze Chimiche e Geologiche, University of Modena and Reggio Emilia, Via Università 4, 41121 Modena, Italy; erika.ferrari@unimore.it; 5 Dipartimento di Biotecnologie, University of Verona, Strada Le Grazie 15, 37134 Verona, Italy

**Keywords:** humic substances, biostimulant, nitrogen, NMR, transcriptomic via RNA-seq, phosphatase, phytase

## Abstract

Lignohumates are increasing in popularity in agriculture, but their chemistry and effects on plants vary based on the source and processing. The present study evaluated the ability of two humates (H1 and H2) to boost maize plant performance under different phosphorus (P) availability (25 and 250 μM) conditions in hydroponics, while understanding the underlying mechanisms. Humates differed in chemical composition, as revealed via elemental analysis, phenol and phytohormone content, and thermal and spectroscopic analyses. H1 outperformed H2 in triggering plant responses to low phosphorus by enhancing phosphatase and phytase enzymes, P acquisition efficiency, and biomass production. It contained higher levels of endogenous auxins, cytokinins, and abscisic acid, likely acting together to stimulate plant growth. H1 also improved the plant antioxidant capacity, thus potentially increasing plant resilience to external stresses. Both humates increased the nitrogen (N) content and acted as biostimulants for P and N acquisition. Consistent with the physiological and biochemical data, H1 upregulated genes involved in growth, hormone signaling and defense in all plants, and in P recycling particularly under low-P conditions. In conclusion, H1 showed promising potential for effective plant growth and nutrient utilization, especially in low-P plants, involving hormonal modulation, antioxidant enhancement, the stimulation of P uptake and P-recycling mechanisms.

## 1. Introduction

Phosphorus (P) is an essential macronutrient that plants require for their growth and metabolism. It serves as a structural component of key biomolecules and plays a vital role in several cellular processes. Plants take up P in the form of orthophosphate ion (inorganic P, Pi) and incorporate it into organic compounds. The interaction of orthophosphate with soil constituents in a broad pH range results in a low concentration of P in the soil solution, typically from 0.1 to 10 μM. This concentration is inadequate for supporting plant growth, metabolism, and production [1,2]. Moreover, a significant portion of the total P in soil (20–80%) exists in organic forms. While these forms have the potential to serve as an essential source of P for plant nutrition, they require enzymatic hydrolysis to become available for plants and are subject to the same soil retention mechanisms as is Pi [3,4,5]. Therefore, P deficiency represents a critical environmental stressor that limits crop productivity [6].

Plants have developed adaptive strategies to cope with P deficiency, which involve increasing P acquisition efficiency (PAE) or P utilization efficiency (PUtE). These strategies include adjustments of root traits and architecture, rhizosphere acidification, the root exudation of organic acid anions, and the formation of biotic rhizosphere associations [7,8,9]. The release of extracellular enzymes, such as phosphatases and phytases, by roots in response to P deficiency has also been shown to improve plant P nutrition [10]. Phytases, in particular, have a dual role in plant P nutrition. They not only increase P acquisition from the soil by breaking down phytate salts, which constitute a significant fraction of the total organic P in soil [11], but also facilitate the recycling of P pools within the plant, as phytates are a major P storage form in various plant tissues [12].

Research on plant nutrition has revealed that a deficiency in P triggers a series of signals, which ultimately lead to changes in the expression of genes and proteins related to mineral nutrition [13,14,15,16]. These changes are ultimately beneficial to a plant because they aim at improving P use efficiency. For example, a recent study on tomato plants found that after just two days of P starvation, 57 genes were upregulated, while only one gene was downregulated, compared to the number of those under the control P conditions. As the duration of P starvation increased to three and four days, the number of differentially expressed genes also increased to 331 and 406, respectively [17].

A number of strategies could be exploited to enhance P mobility in soil and a plant’s capacity to acquire or mine this element more efficiently. Among them, one that is promising entails the use of biostimulants, i.e., products that are derived from biological sources and/or may contain plant growth-promoting microorganisms (PGPMs) [18,19,20]. These products are capable of enhancing plant nutrition, yield and resistance to adverse conditions when applied at low dosages due to their content in bioactive components or when consisting of microorganisms that make the nutrients in soil more available, protect plants from abiotic stress and regulate plant growth through the release of hormone-like substances [21,22].

The updated EU regulation of the European Parliament and of the Council 2019/1009 classifies humic substances (HS), seaweed extracts, protein hydrolysates and microbial inoculants such as mycorrhizal fungi and rhizobacteria, and beneficial elements as biostimulants. In particular, HS, often commercialized as humates, are a significant category of biostimulants that enjoy substantial market dominance. HS offer numerous benefits in agriculture, including enhancements in soil quality, improvements in carbon (C) and nitrogen (N) metabolism in plants, and modifications to root system architecture for better nutrient acquisition [23,24,25]. In addition, HS can ameliorate soil P utilization by plants and mitigate P losses [26,27,28]. Therefore, they play a fundamental role in various biochemical mechanisms and physiological processes at the plant–soil interface, all of which are integral to the growth, development, and overall productivity of plants [24,29].

In recent decades, lignohumates have found diverse applications in environmental technologies, agriculture and industry. They are anionic polymers composed of high- and low-molecular-weight molecules, and exhibit chelating, buffering, and cation exchange properties due to their many carboxylic and phenolic groups that are attached to the aromatic ring. They are produced as by-products of the sulfite process of wood, where the lignin fraction is sulfonated, degraded, and solubilized in water.

Humates derived from various salts of humic acids, including ammonium and potassium humates, are increasingly being produced [30]. Potassium humates, for instance, are widely used as biostimulants to improve a number of soil properties, such as organic matter (OM) content, water retention capacity, soil structure, and microbial activity. They have also been shown to improve the effectiveness of inorganic fertilizers by stimulating plant growth, enhancing yield and quality, increasing nutrient uptake and assimilation, and bolstering plant resilience against stressful conditions, including salt stress [31,32]. However, the chemistry of commercial humates and the physiological effects they can induce in plants can vary greatly depending on factors such as the starting material, extraction processes, and technical approaches used to produce them [30,33]. Furthermore, even if they originate from the same source and are manufactured by the same company, humates, and HS in general, can exhibit significant differences in composition [34], generally due to different extraction procedures and/or processing methods [35], as well as to variations in the decomposition degree of the source material. Thus, it is crucial to characterize in-depth the marketed products and evaluate their efficacy as biostimulants. The current study aims to evaluate the efficacy of two lignohumates with different chemistries in boosting the performance of maize (*Zea mays*, L.) plants grown under different conditions of Pi availability depending on their capacity to regulate Pi acquisition and utilization by plants.

## 2. Results

### 2.1. Characterization of Humates

#### 2.1.1. Spectroscopic Characterization

**Figure 1:**
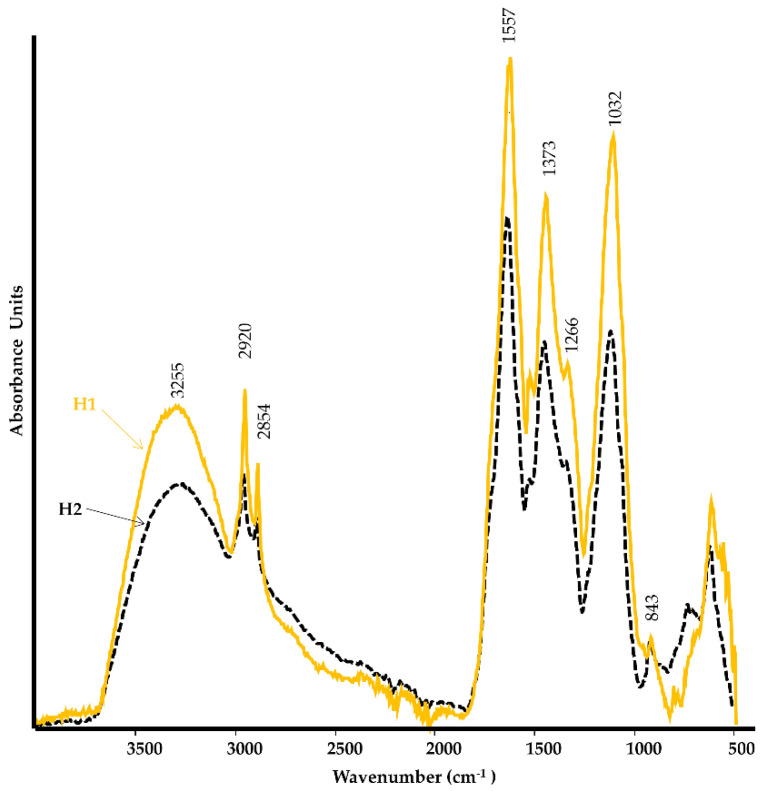
ATR FT-IR spectra of H1 (yellow solid line) and H2 (black dashed line) humates.

The humates exhibited a Fourier transform infrared (FT-IR) spectrum (Figure 1) that is characteristic of HS: a broad band at 3100–3600 cm^−1^ (H-bonded OH and NH groups), twin peaks at 2920 and 2854 cm^−1^ (aliphatic C-H stretching), a band at 1557–1600 cm^−1^ (aromatic C=C stretching, -COO- asymmetric stretching), a shoulder at 1510 cm^−1^ (aromatic C=C stretching), a peak or a shoulder at 1456–1451 cm^−1^ (CH deformation of CH_2_ and CH_3_, and -COO- symmetric stretching), a band at 1393–1373 cm^−1^ (aliphatic CH deformation, -COO- symmetric stretching, and OH deformation), a band at 1266 cm^−1^ (C-O stretching of phenols and ethers, and C-OH deformation), a band at 1170–1030 cm^−1^ (C-O stretching of polysaccharide, C-O-C stretching in ethers, and C-O stretching of polyalcohol), and a band at 836–822 cm^−1^ (C-H deformation in the aromatic ring) [36,37]. The FT-IR spectra of H1 and H2 exhibited a closely similar spectroscopic pattern; however, it is worth noting that the aliphatic component (2800–3000 cm^−1^) appeared more pronounced in H1.

**Figure 2:**
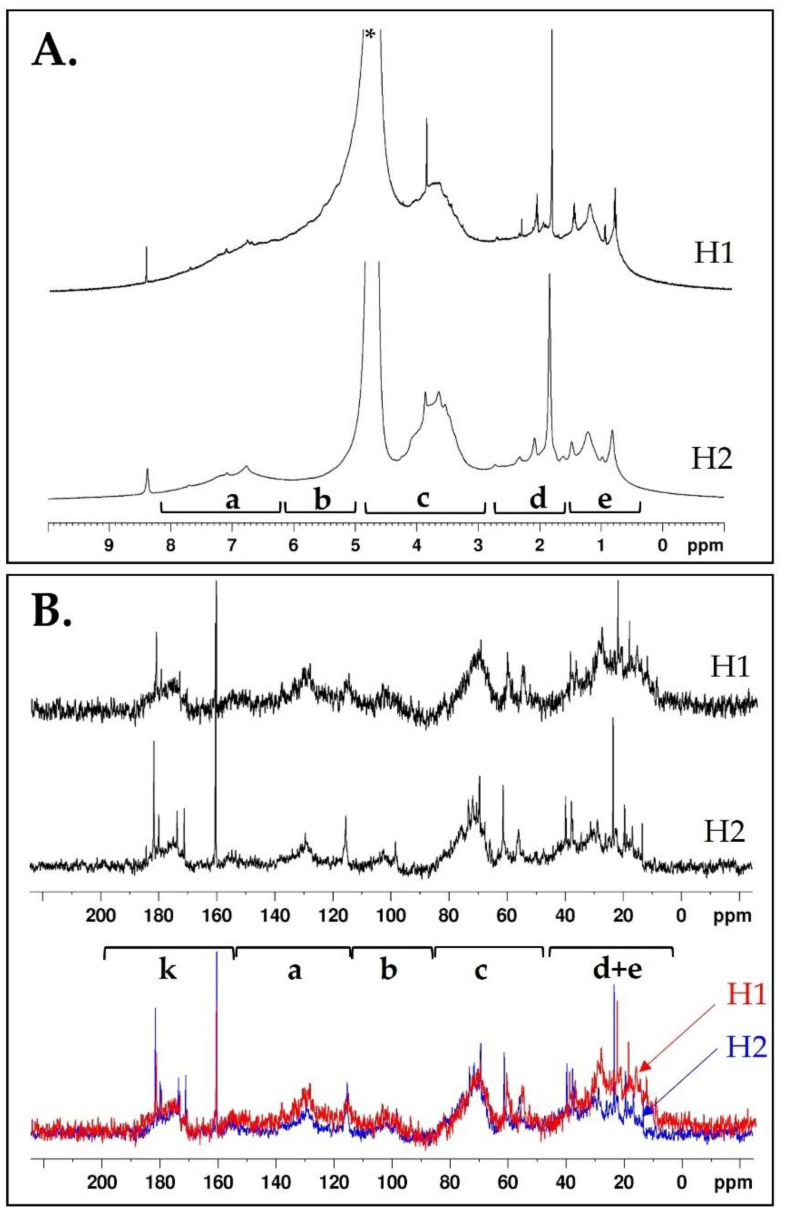
(**A**) ^1^H NMR spectra of humates H1 and H2 in D_2_O at 298 K @ 600 MHz (Bruker pulse sequence: zg, td 32k, ns 128, D1 5s). * Residual HOD. (**B**) ^13^C NMR spectra of humates H1 and H2 in D_2_O at 298 K @ 600 MHz (top) and their overlap (bottom) (Bruker pulse sequence: zgpg_pisp_f2.fas, td 132k, ns 4k, and D1 5s). Typical resonances: (a) aromatics; (b) α-dioxygen and olefins; (c) α-oxygen/nitrogen; (d) β-olefins, β-ether/ester/amine; (e) aliphatics; (k) carbonyl (acid/ester/amide/ketone).

Solution nuclear magnetic resonance (NMR) was chosen over HR-MAS NMR due to the partial solubility of the samples in the selected solvent (D_2_O). Furthermore, the utilization of a cryoprobe significantly enhanced both sensitivity and resolution. As reported in Figure 2, the samples revealed the typical composition of soil OM. Spectral width can be divided into five main regions (a–e) (Figure 2A) [38]. ^1^H NMR signals of both samples results to be quite similar (Figure 2A); however, H1 is characterized by sharper resonances, particularly in the aliphatic region, while H2 shows line-broadening, which suggests the presence of higher-molecular-weight structures. Both samples were rich in carbohydrates and amino acids as pointed out by intense signals in the 3–4.5 ppm region. A methoxyl group is present in both humates (3.85 ppm), as well as acetyl (1.84 ppm). A decrease in chain CH_2_ intensity (1.0–1.3 ppm) in favor of terminal CH_3_ (0.8 ppm) is observed in the order H1 > H2. Aromatic protons are observed especially for sample H2. The formate signal (8.4 ppm) is detected in both humates.

^13^C NMR spectral data (Figure 2B) are commonly divided into six regions associated with different C types [39]: (d/e) 0–45 ppm alkyl C; (c) 46–90 ppm methoxyl and N-alkyl C, and O-alkyl C; (b) 91–110 ppm di-O-alkyl C; (a) 111–160 ppm aromatic C (phenolic and O-aryl C); (k) 161–200 ppm carbonyl C (acid/ester/amide/ketone). As shown in Figure 2B, the two humates present resonances typical of soil OM with broad peaks due to macromolecular structures associated with proteins/carbohydrates and aliphatic species such as waxes. Some sharp and intense peaks related to the presence of low-molecular-weight water-soluble components are also observed in both species. As shown by the overlap of ^13^C NMR spectra obtained by equalizing the integrated area of the signals of region c (Figure 2B, bottom), the area of region d/e is greater for H1 than H2. Aromatic species related to lignin and derivatives are detected in both samples (a broad resonance around 130 ppm), although R2 is slightly bigger for H2 (Table 1). R3 is almost equivalent for both samples (Table 1), suggesting a similar content of carbonyl groups, and is slightly bigger for H1. The ^13^C NMR results are in agreement with the higher C/O ratio in H1.

#### 2.1.2. Elemental Content and Acidity

The two humates displayed differences in elemental composition (Table 1). Specifically, the contents of C, N, and hydrogen (H) were significantly higher in H1 vs. H2, while the opposite occurred for sulfur (S), whose concentration was quite low in both humates, and oxygen (O). The C/N and C/H ratios, generally used as indicators of soil OM stability, averaged to be around 26 and 8, respectively, suggesting a relatively low degree of recalcitrance. Additionally, H1 exhibited lower acidity compared to H2 (Table 1), as also confirmed by the higher C/O ratio.

#### 2.1.3. Thermal Stability

Thermogravimetric curves of humates were characterized via two main steps of weight loss (WL) in the range of temperature investigated; the first was between 250 and 350 °C, which is generally associated with more easily oxidizable compounds, including polysaccharides (e.g., cellulosic material) and aliphatic structures, and the second was between 400 and 500 °C, due to the thermal degradation of more recalcitrant, aromatic structures (e.g., lignin, non-hydrolysable compounds) [40,41,42,43]. Thermal analysis suggested a higher stability of H2 compared to that of H1, as proven by the higher values of the ratios between WL occurring at high vs. low temperature ranges (WL_400–550/200–300_ and WL_400–550/300–400_) and of the temperature at which half of the OM was lost (TG-T_50_) (Table 1). This agrees with ^13^C NMR data, reporting a higher presence of aromatic species related to lignin and derivatives in H2.

#### 2.1.4. Hormone Quantification

All tested hormones, namely indoleacetic acid (IAA), zeatin riboside (ZR), gibberellic acid (GA), and abscisic acid (ABA), were detected in both humates (Table 1). However, there were significant differences (*p* < 0.05) in the contents of individual hormones between the two humates. While the IAA content was similar between H1 and H2, ZR content was higher in H1 than in H2. On the other hand, ABA content was higher in H1 than in H2 although differences were not significant, while the GA content showed a unique trend, with H2 having higher content than H1 did. ABA and cytokinins were found to be more abundant than auxins and GA were in both humates.

#### 2.1.5. FRAP and Phenol Content

The antioxidant capacity measured in terms of ferric-reducing antioxidant power (FRAP) and the content of total phenols (TP) significantly differed between humates (Table 1). In particular, the FRAP was about 7.4-fold higher in H1 than that in H2. Similarly, the content of total phenols was 1.5-fold higher in H1 than that in H2.

### 2.2. Effects of Humate Application on Plant Growth, SPAD, C and N Contents and FRAP

**Figure 3:**
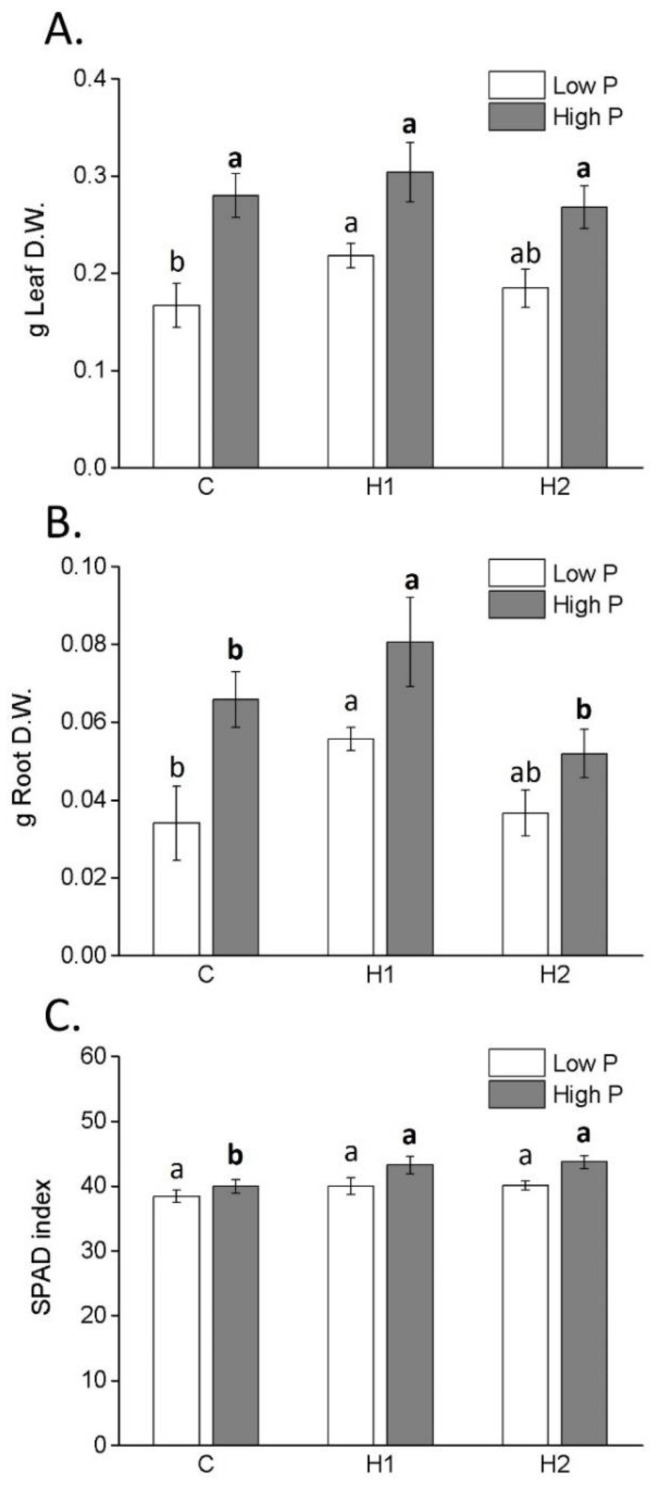
Dry weight (D.W.) of leaves (**A**) and roots (**B**), and SPAD index (**C**) (mean ± SD) of maize plants treated or not treated with humates H1 and H2. Different letters above bars indicate significant differences between treatments (*p* < 0.05).

The application of H1 significantly enhanced the leaf and root biomass production of maize plants that received low P (Figure 3A,B). On the contrary, H2 did not show any such effect on the plants.

Differences in the SPAD index between the plants were more pronounced in the high-P condition (Figure 3C). In this case, both H1 and H2 were found to be effective in increasing the SPAD index relative to that of the control.

Both humates increased the percentage of N in roots under low-P and high-P conditions (Table 2). In leaves, N accumulation was found to be more enhanced after the application of H1 to the plants under either P condition. The C content in roots did not show any significant variation between the control and treated plants under low P, while a trend towards an increase of this element was observed in plants treated with the individual products under high P. The leaf C content was not influenced by humates under either P dose.

The antioxidant activity measured using the FRAP assay was higher in plants treated with H1 compared to that of untreated plants and of those treated with H2 (Table 3).

### 2.3. P accumulation, PAE and Activity of P-Mining Enzymes

**Figure 4:**
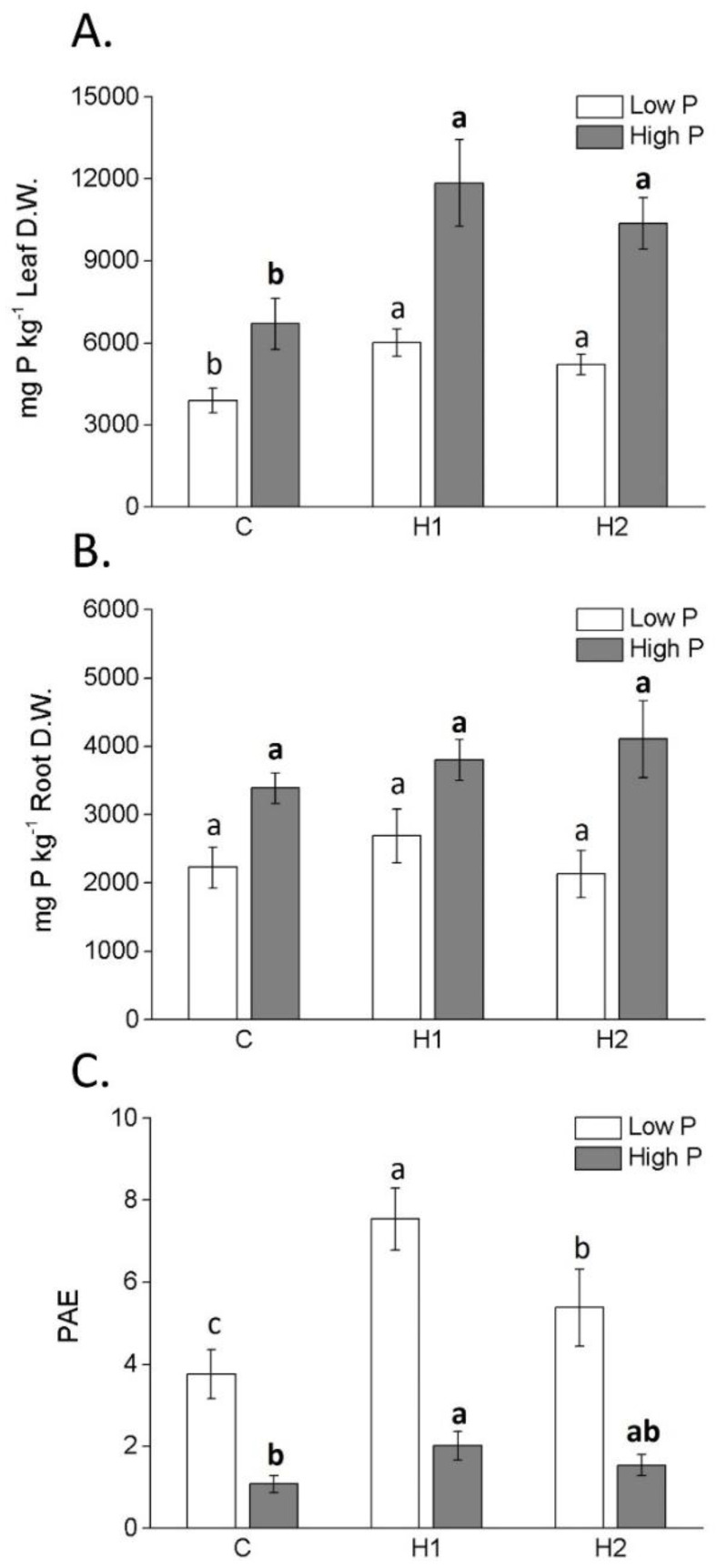
Phosphorus concentration in leaves (**A**) and roots (**B**), and phosphorus acquisition efficiency (PAE) (**C**) (mean ± SD) of maize plants treated or not treated with humates H1 and H2. Different letters above bars indicate significant differences between treatments (*p* < 0.05).

When plants received a high P supply, the concentration of P in their leaves was mostly increased by both humates compared to that of the control plants (Figure 4A), while under low-P treatment this effect was only triggered by H1.

No variation in root P content was observed between humate-treated plants and the control (Figure 4B). Phosphorus acquisition efficiency (PAE) was higher in plants treated with humates than in the untreated plants irrespective of the P supply (Figure 4C). In addition, values were more elevated under low-P than high-P conditions. Maximum PAE values under high P were associated with plants treated with H1.

**Figure 5:**
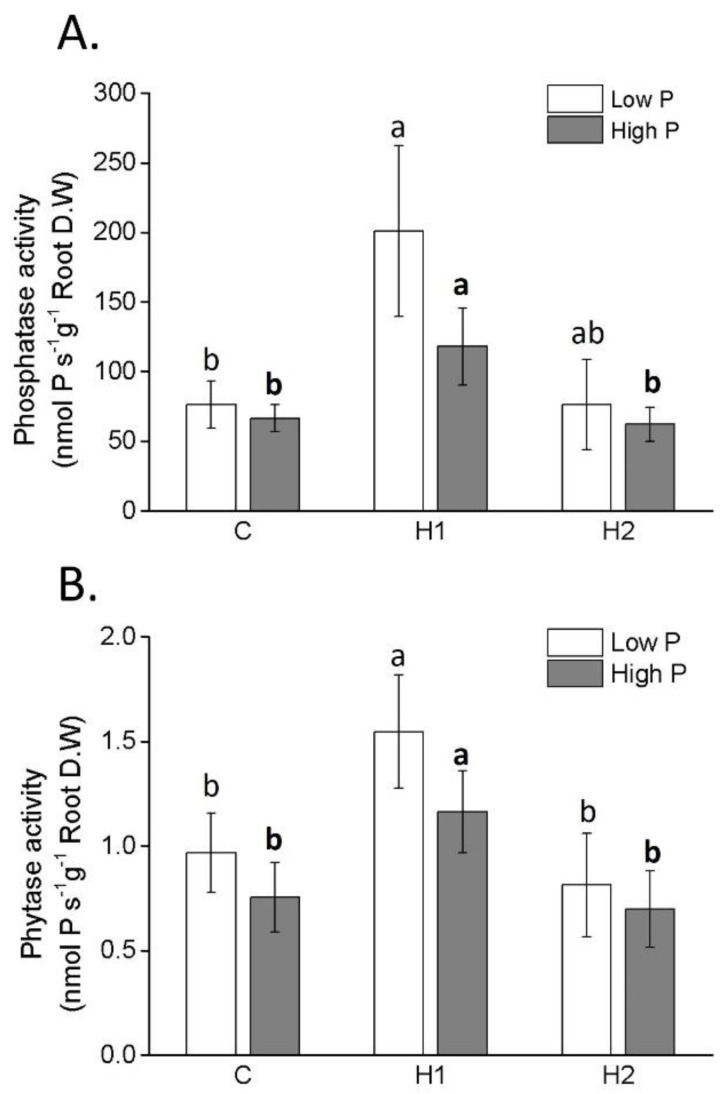
Phosphatase (**A**) and phytase (**B**) activities (mean ± SD) in roots of maize plants treated or not treated with humates H1 and H2. Different letters above bars indicate significant differences between treatments (*p* < 0.05).

The activity of P-mining enzymes in the roots of maize plants is reported in Figure 5A,B. The treatment of plants with H1 promoted phosphatase activity compared to that of the control plants, under high and low P, while H2-treated plants showed no enhanced activity. The effect ascribed to H1 was more evident in plants grown under low P, although differences were not significant. The activity of phytase was substantially lower than of that of phosphatase, and was more pronounced under low-P than high-P conditions. As in the case of phosphatase, maximum values were recorded in the roots of plants treated with H1, under both P treatments. No stimulatory effect of phytase activity was observed in plants following H2 application.

### 2.4. Spectroscopic Characterization of Maize Leaves

The analysis was conducted on maize plants treated with H1 and untreated plants. All leaf samples showed a similar FT-IR absorption spectrum (Figure S1) in the following spectral range: a broad band at 3400–3000 cm^−1^ (H-bonded OH), bands at 3000–2800 cm^−1^ (aliphatic C-H stretching), two strong peaks at 1643–1632 cm^−1^ (C=O stretching of amide I and quinone), at 1564–1560 cm^−1^ (Amide II, aromatic C=C stretching, and -COO- asymmetric stretching), a peak of variable intensity at 1373 cm^−1^ (aliphatic CH deformation, -COO- symmetric stretching, and OH deformation), a weak band at 1258 cm^−1^ (Amide III, C-O stretching of phenols and ethers, and C-OH deformation) and a very strong band at 1035–1033 cm^−1^ (C-O stretching of polysaccharide, and C-O-C stretching in ethers) [44].

In contrast, the leaf sample derived from plants grown under high P and treated with H1 (Figure S1) was characterized by more intense bands at 3289 cm^−1^ (H-bonded OH) and 1643 cm^−1^ (C=O stretching of amide I and quinone) compared to the leaf sample derived from control plants. On the other hand, the latter was distinguished from the former by the higher intensity of the band at 1564 cm^−1^ (Amide II, aromatic C=C stretching, and -COO- asymmetric stretching) and 1372 cm^−1^ (aliphatic CH deformation, -COO- symmetric stretching, and OH deformation).

### 2.5. Changes in Gene Expression as a Function of P Supply and H1 Treatment

Given that H1 demonstrated more pronounced positive effects on maize plants in terms of growth stimulation, nutrition, and antioxidant capacity, we focused on the transcriptomic analysis on leaves of untreated plants and plants treated with this specific humate. By doing so, we aimed to gain deeper insights into the molecular mechanisms and pathways that underlie these significant effects observed in the presence of H1. We conducted a comparison between the gene expression profiles of plants subjected to H1 treatment under high- and low-P conditions, and those of the control plants. For simplicity, we have selected those differentially expressed genes (DEGs) showing more interesting changes in terms of the stimulation of plant metabolism, growth and regulatory mechanisms. Furthermore, we also compared the gene expression profiles of plants grown under high- and low-P conditions, regardless of the H1 treatment, to investigate the impacts of P deficiency. For each comparison, the number of DEGs is reported in Table 4.

**Figure 6:**
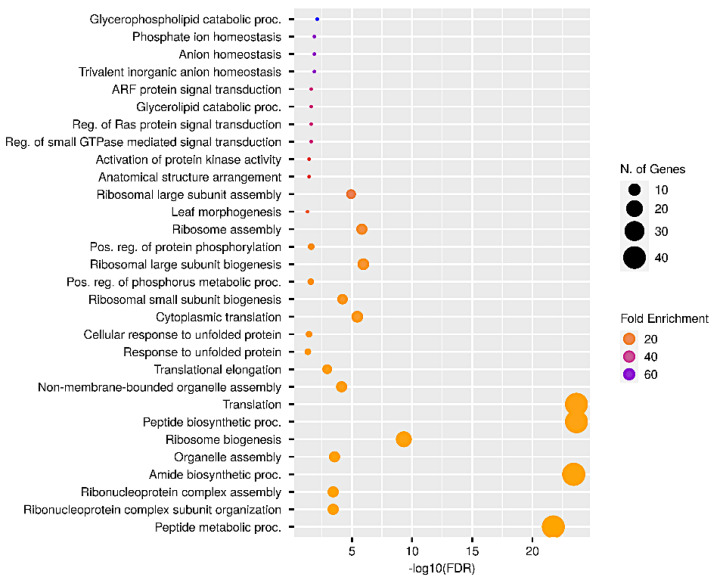
Significantly enriched GO biological process terms (FDR ≤ 0.05) in DEGs of plants grown under low P compared to high P. GO terms are sorted in accordance with their fold enrichment along the *y* axis, whereas the *x* axis represents the level of significance (−log10(FDR)).

The DEGs obtained from the comparison between LP and HP conditions with very-many-fold enrichment were mainly related to the biological processes of glycerophospholipid catabolism (GO:0046475), phosphate ion homeostasis and anion homeostasis (GO:0055062; GO:0055081), ribosome assembly and translation (GO:0042255; GO:0006412), (trivalent) inorganic anion homeostasis (GO:0030002), ARF protein signal transduction (GO:0032011), ribosome assembly and translation (GO:0042255; GO:0006412), glycerolipid catabolic processes (GO:0046503), the regulation of Ras protein signal transduction (GO:0007265), the regulation of small GTPase-mediated signal transduction (GO:0007264), the activation of protein kinase activity (GO:0032147) and anatomical structure arrangement (GO:0048532) (Figure 6). The most significantly enriched GO terms and the complete list of DEGs obtained from the three comparisons is available in Table S1.

In P-starved plants, the expression levels of key genes encoding proteins known to be induced by phosphate deficiency were significantly higher compared to those of P-sufficient plants (Table 5).

These genes included candidates such as putative glycerol-3-phosphate transporter 1, putative 1-acyl-sn-glycerol-3-phosphate acyltransferase 4, phosphatase phospho 1, and inositol-1-monophosphatase. Furthermore, under the low-P condition, genes encoding proteins involved in establishing symbiotic interactions with rhizobionts, including the rhicadhesin receptor, were upregulated. Additionally, genes associated with stress signaling, such as putative peptidyl-prolyl cis-trans isomerase, WD40 repeat domain family protein, and Bax inhibitor 1 exhibited increased expression. 0The low-P condition also led to the upregulation of genes involved in hormone signaling, such as protein BRASSINAZOLE-RESISTANT 1, phosphoinositide signaling, such as that of SEC14-like protein 1, and plant growth, including that of SAUR56-auxin-responsive SAUR family member and protein OBERON 3.

Under low-P conditions, the comparison between untreated plants and plants treated with H1 (LP vs. LP_H1) showed an increase in the expression of genes involved in hormone homeostasis and synthesis such as cytokinin-O-glucosyltransferase 3 and Protein ABA DEFICIENT 4 (chloroplastic). Moreover, genes associated with transcriptional control (e.g., AT-hook motif nuclear-localized protein 26), iron sensing (e.g., nicotianamine synthase 3), peroxisome biogenesis and organization (e.g., peroxisomal biogenesis factor 19), phosphate recycling activity (e.g., nudix hydrolase 13), vitamin biosynthesis (e.g., biotin synthase and pyridoxal phosphate (PLP)-dependent transferase superfamily protein), defense against stress (e.g., WRKY22 and MATH domain-containing protein), chlorophyll biosynthesis (e.g., protochlorophyllide reductase B), cellular redox homeostasis (e.g., grx_S12-glutaredoxin subgroup I, grx_S16-glutaredoxin subgroup II), signal transduction (e.g., ran-binding protein 1), reproduction (e.g., nucleoredoxin 1), and transcriptional regulation (e.g., zinc finger CCCH domain-containing protein 15 homolog, Dof zinc finger protein DOF2.2, Transcription factor TCP14) were upregulated in response to H1 treatment. In addition to these genes, there were others that exhibited increased expression levels. These included genes associated with fatty acid biosynthesis and lipid metabolism (e.g., 12-oxophytodienoate reductase 8; Oxysterol-binding protein-related protein 2A; Lipase-like), peptide transport (e.g., oligopeptide transporter 3) and plant growth (e.g., growth-regulating-factor-interacting factor 1).

Under high-P conditions, we noted that the treatment of plants with H1 resulted in the upregulation of genes associated with S transport and metabolism. Notably, these genes included sulfate transporter 6, methionine adenosyltransferase, and methionine aminopeptidase. Furthermore, H1 treatment induced the upregulation of genes involved in growth-promoting hormones, particularly those implicated in the regulation of leaf size, cell wall formation, and root hair elongation. These genes included auxin response factors such as auxin response factor 10 and auxin response factor 21, putative LRR receptor-like serine/threonine-protein kinase, SNARE-interacting protein KEULE, SCARECROW-like protein, WAT1-related protein, and somatic embryogenesis receptor-like kinase 3. Genes involved in sugar metabolism, namely trehalose-6-phosphate synthase, alkaline alpha galactosidase 3, and probable galactinol--sucrose galactosyltransferase 1, as well as genes associated with plant defense, such as 60 kDa jasmonate-induced protein and LRR receptor-like serine/threonine-protein kinase, showed upregulation upon H1 treatment. Similarly, genes involved in signal transduction pathways, including putative glycogen synthase kinase family protein and Anamorsin homolog, and genes related to protein folding, such as chaperone DNA J2, and transcriptional regulation, including Zinc finger CCCH domain-containing protein 19, DNA-directed RNA polymerase subunit beta’-like, and CBF1-interacting co-repressor CIR N-terminal, exhibited higher expression levels in H1-treated plants.

## 3. Discussion

This study underscores substantial differences in the chemical and biological properties of the two humates examined as potential biostimulants. Their molecular complexity was thoroughly investigated via a comprehensive set of analyses. Specifically, H2 exhibited higher aromaticity and a higher molecular weight, as well as higher thermal stability, thus suggesting greater OM recalcitrance. In contrast, H1 displayed signs of greater degradation, resulting in lower-molecular-weight species, and a relatively higher content of aliphatic molecules. Therefore, H1 seems to have greater reactivity compared to that of H2, which is in line with the observation that organic substances displaying signs of degradation or with a low molecular weight tend to be more reactive and able to interact with membrane receptor systems by transmitting signals to intracellular effector structures. Despite these distinctions, both humates similarly improved N content in maize plants, irrespective of the P concentration applied, indicating their effectiveness in enhancing N acquisition. This finding aligns with previous research acknowledging HS’ role in upregulating nitrate influx transporters at root cell membranes [45]. The increase in N accumulation in plants treated with HS was consistent with the increase in the SPAD index, but this relationship was significant only under adequate P supply. The SPAD index, calculated using a widely utilized chlorophyll meter in agricultural systems, guides N management via monitoring the leaf N status [46]. The humate effect on N content without a SPAD index changes under low-P conditions and can be explained via a consideration of the impact on photosynthesis. Scarce P availability can hinder photosynthesis, affecting ATP and NADPH formation in light reactions [47,48]. To counter excessive accumulation of reactive oxygen species (ROS) resulting from reduced electron flux in photosystems, plants control chlorophyll synthesis and breakdown, often lowering their content [49]. Plants could eventually allocate more N towards the production of other N-containing compounds. In our study, the transcriptomic analysis revealed the upregulation of the gene coding for protochlorophyllide reductase B in P-starved H1-treated plants. This enzyme is crucial in the later steps of chlorophyll biogenesis [50]. Its upregulation suggests that H1 treatment stimulated chlorophyll biosynthesis in P-depleted plants, possibly due to increased P accumulation. Therefore, we hypothesize that the amount of chlorophyll produced in P-starved H1-treated plants may not have been substantial enough to discern noticeable differences when compared to the untreated control. In addition, chlorophyll degradation processes might have balanced chlorophyll levels in P-starved plants and could have potentially offset any significant increases in chlorophyll content.

Beyond boosting N content in plants, the two humates increased P leaf accumulation of both P-sufficient and P-limited plants. Notably, while there were no observable changes in P accumulation within the roots, it is plausible to hypothesize that plants treated with humates efficiently translocated P from the roots to the shoots compared to the untreated plants. Furthermore, despite higher PAE values in all P-starved plants than in P-sufficient ones, the addition of H1 led to a remarkable 2-fold and 1.5-fold PAE increase compared to the untreated plants and those receiving H2, respectively, regardless of the P applied condition. This finding suggests that H1 outperformed H2 in triggering maize plant responses to P shortage, thereby promoting efficient P uptake. The observed effect could be possibly attributed to root phosphate transporter modulation, as found by Jindo et al. [26] in tomato plants.

Consistent with these findings, we noted higher activity of phosphatase and phytase enzymes in maize roots when plants were treated with H1 compared to the other two groups. These enzymes have a key role in P mining in soil [51], and in internal P recycling [52,53]. Their activity typically increases under P shortage, as reported in previous studies [52,53]. However, after the application of either humates, as well as in the untreated plants, no significant differences in the activity of these enzymes were evident between P-sufficient and P-starved plants. In light of this, it becomes clear that the activity of the two enzymes was only enhanced by H1, whereas either the low concentration of P applied and H2 failed to elicit this effect. The impact of HS on extracellular phosphatases has been documented [54], but the specific mechanism effecting intracellular phosphatase and phytase activity still remains unclear. Most likely this effect occurs, at least in part, via mechanisms operating at transcriptional level. Indeed, we did notice upregulation of genes encoding for phosphatase phospho 1 and inositol-1-monophosphatase in leaves of the P-starved plants. Moreover, the addition of H1 to P-starved plants induced the upregulation of a gene coding for the nudix hydrolase 13, involved in P recycling via pyrophosphatase activity [55]. These results are consistent with the increase of PAE in maize plants supplied with low P and H1. It is important to highlight that while the transcriptomic analysis was focused on leaves, the impact of H1 on phosphatase-encoding genes may extend to the roots as well, potentially correlating with enzyme activity.

The remarkably positive effect of H1 on N, P, PAE, phosphatase and phytase enzymes, and on genes involved in P recycling under P-deficiency led to significantly greater leaf and root biomass production compared to both untreated plants and those treated with H2. This finding highlights the promising potential of H1 as a highly effective promoter of plant growth and nutrient utilization, particularly in P-limited conditions, which is in line with several studies reported in the recent review by Jindo et al. [56]. HS function as carriers for phytohormones and hormone-like molecules, including derivatives of phytohormonal compounds. In the rhizosphere, HS undergo mild hydrolysis, releasing these entrapped compounds that further bind to root cell membrane receptors [24,56]. This interaction initiates a cascade of responses in plants, notably affecting growth. In particular, the plant hormone signaling pathways are primary targets of HS and substantially affect plant developmental processes [24,57]. HS mainly target auxins, crucial regulators of cell division and elongation [58]. However, it is worth noting that HS impact extends beyond just auxins, as they can also modulate other hormones in plants, including ABA [59], ethylene, and nitric oxide [60]. These hormones, in concert with auxins, regulate not only root growth, but also various traits associated with nutrient acquisition [61]. Consistently with previous literature about the effect of HS on the plant hormone signaling network, our study revealed upregulation of several genes involved in hormone signaling in H1-treated plants. For instance, in P-starved plants, genes coding for cytokinin-O-glucosyltransferase 3 and Protein ABA DEFICIENT 4 were upregulated, indicating the potential modulation of cytokinin and ABA signaling pathways. On the other hand, in P-sufficient plants, genes coding for auxin response factor 10, auxin response factor 21, auxin-responsive SCARECROW-like protein, and WAT1-related protein (a vacuolar auxin transporter) were upregulated, pointing towards the influence of auxin signaling. Furthermore, our analysis highlighted the upregulation of several genes associated with growth and developmental processes, such as growth-regulating-factor-interacting factor 1 and somatic embryogenesis receptor-like kinase 3.

Both H1 and H2 contained traces of various endogenous phytohormones, including auxins, ABA, cytokinins (zeatin riboside), and GA, but in different concentrations. Specifically, H2 contained comparable levels of endogenous auxins and ABA as H1, but lower levels of cytokinins (zeatin riboside) and higher GA content. These differences in hormone composition between H1 and H2 could partly explain their distinct capacities to stimulate plant growth, as reported in previous studies [62]. Indeed, the dynamic interplay between auxins and other hormones is well documented in orchestrating plant growth and development in response to changing environmental conditions [61]. A number of studies consistently found auxins in HS, with some demonstrating the hormone-like activities of HS [63,64,65]. Moreover, research has suggested that variations in hormone activity in HS can account for varying degrees of stimulation in plant growth [62].

Another significant positive effect of H1 on maize plants was its ability to enhance antioxidant capacity in both leaves and roots. As for H2, a similar effect was observed, but only in leaves and specifically when the plants were experiencing P starvation. The enhancement of antioxidant defenses in plants is a characteristic shared by many biostimulants, including HS [63,66]. The content of phenols and FRAP activity were both higher in H1 than H2, indicating that H1 was richer in molecules that exhibit antioxidant activity. Phenol compounds in low quantities in the external environment, in particular, can trigger responses in plants that enhance their defense mechanisms against various stress conditions [67]. In addition to stimulate antioxidant plant defense, they can serve as signaling molecules that stimulate growth processes, including root development, and nutrient uptake [67]. Previous studies demonstrated that HS primarily target the phenylpropanoids biosynthesis pathway, which his important for the production of antioxidant compounds involved in plant defense. These compounds bolster a plant’s capacity to respond to abiotic stresses and protect against various threats [63]. Similar to other abiotic stresses, P limitation can induce oxidative stress in plants [68]. Consequently, adding either H1 or H2 likely aids in ROS scavenging and enhances plants resilience to the nutrient constraint. H1, in particular, showed a greater capacity than H2 did in inducing this beneficial effect. Consistent with this finding, H1 upregulated defense genes, both under a low and high P supply, which is notably linked to redox balance under P shortage. Several other defense genes showed upregulation. Overall, these results highlight the potential role of H1 in enhancing a plant’s ability to cope with oxidative stress when P availability is limited.

## 4. Materials and Methods

### 4.1. Humate Characterization

This study was carried out testing two non-marketed humates (designated as H1 and H2) which were provided by the company NEOVA (Jyväskylä, Finland).

#### 4.1.1. FT-IR and NMR Spectroscopy

The FT-IR spectra of humates H1 and H2 were recorded using a Bruker Tensor FT-IR instrument (Bruker Optics, Ettlingen, Germany) equipped with an accessory for analysis under micro-attenuated total reflection (ATR). The sampling device contained a microdiamond crystal, and a single reflection with an angle of incidence of 45° (Specac Quest ATR, Specac Ltd., Orpington, Kent, UK). Spectra were recorded from 4000 to 400 cm^−1^, with a spectral resolution of 4 cm^−1^ and 100 scans. Background spectra were acquired against air under the same conditions prior to each sample. Spectra were processed with the Grams/386 spectroscopic software (version 6.00, Galactic Industries Corporation, Salem, NH, USA). The freeze-dried sample amount used was smaller than 1 mg.

NMR spectra were recorded using a Bruker Biospin FT-NMR AVANCE III HD (600 MHz) spectrometer equipped with CryoProbe BBO H&F 5 mm under inverse detection. The nominal frequencies were 150.90 MHz for ^13^C and 600.13 for ^1^H. For each sample, 10 mg was suspended in 0.6 mL of D_2_O (99.8%) into a 5 mm NMR tube. A 90° pulse was calibrated for each sample and standard NMR parameters were used. Briefly, 1D ^1^H and ^13^C NMR spectra were acquired using typical Bruker pulse sequences (i.e., zg, and zgpg_pisp_f2). Three ratios of integrated areas were calculated from ^13^C NMR spectra, namely R1 = area(d + e)/area(c); R2 = area(a + b)/area(c + d + e); R3 = area(k)/area(c + d + e).

#### 4.1.2. Elemental Analysis and Total Acidity

The concentration of total C, N, H and S was determined via dry combustion using a CHNS macro-analyzer (vario Macro cube, Elementar, Langenselbold, Germany). Samples were analyzed in quintuplicate. The concentration of O was determined via difference. Alfalfa OAS (B2273, Elemental Microanalysis Limited, Okehampton, UK) was used as reference material. QA/QC values were 98.8% for C, 100.2% for N, 97.7% for H and 102.9% for S. Total acidity given by the sum of COOH and acidic (phenolic) OH groups was determined in accordance with the method proposed by Schnitzer and Gupta [69] via equilibration with Ba(OH)_2_ and via discontinuous titrations.

#### 4.1.3. Thermal Analysis

The nature and biochemical recalcitrance of humate samples were assessed using a thermogravimetric (TG) analyzer coupled with simultaneous differential scanning calorimetry (DSC) (TGA-DSC 3+, Mettler Toledo, Switzerland). An aliquot of ca. 30 mg of each humate was placed in an alumina crucible and heated from 30 to 700 °C at 10 °C min^−1^ in air at a flow rate of 100 mL min^−1^. Ratios between weight losses (WL) occurring within different temperature ranges and the temperature at which half of biomass was lost (TG-T_50_) were then calculated.

#### 4.1.4. Hormone Quantification

Hormone levels in humates were measured by extracting them in water at a concentration of 50 mg per 100 mL. The concentration of IAA, ZR, ABA and GA was determined using a competitive inhibition enzyme immunoassay technique. Specifically, the Mini Sample Enzyme-Linked Immunosorbent Assay Kit (ELISA, My Bioresource) was used to quantify indoleacetic acid (IAA) levels, with a pre-coated microplate containing an antibody specific to IAA. Biotin-labeled IAA was used as a standard, and unlabeled IAA was used as a sample. Avidin conjugated to horseradish peroxidase (HRP) was added to each well, and the concentration of IAA in the sample was determined by measuring the bound HRP conjugate at λ = 450 nm. IAA standards ranging from 0 to 200,000 pg mL^−1^ were used.

Zeatin riboside (ZR) levels in humates were measured using an ELISA kit (My Bioresource), which included a microliter plate pre-coated with an antibody specific to ZR. Samples, along with horseradish peroxidase (HRP)-conjugated ZR, were added to the plate wells and incubated. The color intensity was detected at λ = 450 nm using a microplate reader. ZR standards ranging from 0 to 80 ng mL^−1^ were used.

Abscisic acid (ABA) in humates was quantified using the ELISA kit (My Bioresource), with a pre-coated plate containing a plant ABA antibody. ABA in the sample bound to the antibodies coated on the wells. Biotinylated plant ABA antibody was then added and bound to ABA in the sample, followed by Streptavidin-HRP which was bound to the biotinylated plant ABA antibody. The color developed in proportion to the amount of ABA, and the reaction was terminated via adding an acidic stop solution. Absorbance was measured at λ = 450 nm using a microplate reader. ABA standards ranging from 0 to 160 ng mL^−1^ were used.

Gibberellic acid (GA) in humates was quantified using an ELISA kit (My Bioresource), with triplicate measurements. The plate was pre-coated with plant GA antibody, and GA in the sample bound to the antibodies on the wells. Biotinylated plant GA antibody was added and bound to GA in the sample, followed by Streptavidin-HRP which bound to the biotinylated plant GA antibody. Unbound Streptavidin-HRP was then washed away, and a substrate solution was added, leading to color development in proportion to the amount of GA. The reaction was terminated by adding an acidic stop solution, and absorbance was measured at λ = 450 nm using a microplate reader. GA standards ranging from 0 to 640 pmol L^−1^ were used in the assay.

Analyses of hormones for each humate were conducted in triplicate.

#### 4.1.5. Analysis of Antioxidant Capacity (FRAP = Ferric-Reducing Antioxidant Power Assay) and Total Phenols

Total antioxidant activity was evaluated in three replicates of each humate, as well as in the leaves and roots of maize plants, using the ferric-reducing antioxidant power assay (FRAP), based on the methodology of Benzie and Strain [70]. The concentration of total phenols in each humate sample was determined in triplicate using the Folin–Ciocalteau (FC) assay with gallic acid as the calibration standard using a Shimadzu UV-1800 spectrophotometer (Shimadzu Corp., Columbia, MD, USA). Humates were extracted and treated in accordance with the protocol by Nicoletto et al. [71]. The absorbance of samples was measured at λ = 765 nm. Results were calculated from a standard curve of gallic acid concentrations ranging from 0 to 600 mg mL^−1^ and expressed in milligrams of gallic acid equivalent per kilogram of fresh weight.

### 4.2. Plant Growth and Experimental Design

Seeds of *Zea mays* L. were soaked in distilled water overnight and then surface-sterilized with 5% (*v*/*v*) sodium hypochlorite. After germinating on filter paper, seedlings were transferred to 3 L pots with thoroughly aerated ¼ Hoagland nutrient solution [72] with a density of 10 plants per pot. The nutrient solution contained 250 μM KH_2_PO_4_. Plants were grown inside a chamber with 14 h of light per day, an air temperature of 21 °C (night) and 27 °C (day), a relative humidity of 70/85%, and a photon flux density of 280 mol m^−2^ s^−1^. Twelve-day-old plants were then divided in two groups, one receiving 250 μM KH_2_PO_4_ (high P), and the other receiving 25 μM KH_2_PO_4_ (low P). Plants under high and low P were further divided in subgroups, each being treated with a single humate through a unique application to the nutrient solution at 1 mg C L^−1^. Plants not treated with humates served as controls. Three pots were setup for each treatment, as well as for the control.

After a four-day period following the addition of humates, the chlorophyll content of the plants was assessed using a non-destructive technique that measures light transmission across a leaf at two different wavelengths. This technique provides an index of chlorophyll content, also known as the SPAD index, via a calculation of the ratio of transmission at the two wavelengths. The analysis was conducted using a SPAD chlorophyll meter (model SPAD-502, Minolta Camera Co. Ltd., Osaka, Japan) on the last fully expanded leaf of maize plants. Determination was carried out for 10 plants per pot for each treatment.

At the end of the treatment, the plants were harvested randomly, washed carefully and dried using blotting paper. A sub-sample of plant material was immediately frozen using liquid nitrogen and stored at −80 °C for future biochemical and molecular analyses. Four plants per treatment per pot were randomly selected, and their fresh weight was recorded. The samples were then placed in a drying oven for 2 days at 70 °C and allowed to cool for 2 h inside a closed bell jar. The dry weight of the individual roots and leaves was then measured for each plant.

FT-IR spectroscopy and a FRAP assay were performed on leaves in accordance with protocol described for humates.

### 4.3. Phosphorus Acquisition Efficiency (PAE)

The P concentration in roots and leaves was determined colorimetrically on dry plant material (50 mg) after sulfuric–perchloric digestion using the malachite green method [73]. The analysis was conducted in triplicates where each biological replicate consisted of one plant per treatment per pot. Phosphorus acquisition efficiency (PAE) was calculated as the ratio of P accumulated in tissues to that exogenously supplied P [52].

### 4.4. Enzyme Activity

Phosphatase and phytase activities were determined in triplicates, where each biological replicate consisted of one plant per treatment per pot, as described in Hayes et al. [3]. Root material was ground in 15 mM 2-(N-morpholino)ethanesulfonic acid (MES) buffer (pH 5.5) containing 0.5 mM CaCl_2_·H_2_O and 1 mM EDTA. The extract was centrifuged at 13,800× *g* for 15 min at 4 °C and the supernatant was gel-filtered at 4 °C on Sephadex G-25 columns. To assay total acid phosphatase activity, the enzyme extract was incubated at 26 °C in 15 mM MES buffer (pH 5.5) with 1 mM EDTA, 5 mM cysteine and 10 mM p-nitrophenyl phosphate (pNPP). The reaction was stopped after 30 min via the addition of 0.25 M NaOH. The concentration of p-nitrophenol (pNP) was determined by measuring the absorbance at 412 nm against that of standard solutions. Phytase activity was measured on the same root extracts and under the same conditions described above, except that pNPP was replaced with 2 mM potassium myoInsP6. The reaction was stopped after 60 min via the addition of ice-cold 10% trichloroacetic acid (TCA), and Pi concentration was determined via the malachite green method [73].

### 4.5. Transcriptomic Analysis and Gene Ontology (GO) Analyses

mRNA was isolated directly from the leaf samples of three biological replicates (1 replicate = 1 plant per treatment per pot) using the Dynabeads mRNA Direct Micro Kit (Thermo Fisher Scientific, Carlsbad, CA, USA) following the manufacturer’s instructions. Poly(A) RNA was used to prepare the sequencing library using Ion Total RNA-Seq Kit v2 (Thermo Fisher Scientific). Following validation, normalization and pooling, libraries were sequenced on an Ion Torrent S5 platform (Thermo Fisher Scientific). Raw RNA-Seq reads were processed to remove short and low-quality reads (phred-like Q value ≤ 20) and they were mapped to the *Zea mays* reference genome (B73 RefGen_v4, available in NCBI) using Bowtie2 (v2.4.2) [74]. The raw reads were checked and processed with Samtools (v1.11) [75] and read counts were obtained for every gene using BEDTools multiBamCov [76]. Genes with an overall expression value lower than 20 were removed. To carry out the differential expression analysis, DESeq2 R package (v.1.32.0) [77] was used and differentially expressed genes (DEGs) were selected considering a *p*-value of <0.05 and a |log2-fold change| ≥ 1.0. The gene ontology (GO) enrichment analysis was performed for genes significantly differentially expressed at a *p*-value of ≤0.01 using the graphical tool ShinyGO 0.76 (http://bioinformatics.sdstate.edu/go76/ (accessed on 30 June 2023)) with a 0.05 FDR cutoff.

### 4.6. Statistics and Bioinformatic Analysis

An analysis of variance (ANOVA) was performed using the SPSS software version 19.0 (SPSS Inc. 1999, Cincinnati, OH, USA) and was followed by pair-wise post hoc analyses (a Student–Newman–Keuls test) to determine which means differed significantly at *p* < 0.05 (±SD). Statistical analyses of RNA-seq data were performed using RStudio (version R-4.1.0).

## 5. Conclusions

Both humates improved maize N content regardless of the P concentration applied, indicating their biostimulant potential. This aligned with the increased SPAD index, notably when P supply was sufficient. Low-P conditions might slow down photosynthesis, affecting chlorophyll production and SPAD index differences. Humates, and especially H1, improved P accumulation in leaves via more efficient root-to-shoot P translocation. H1 outperformed H2 in prompting the maize response to P deficiency, enhancing P uptake and PAE. While both humates heightened phosphatase and phytase enzyme activity in maize roots, H1 showed a more significant effect. In particular, its greater positive influence on N, P, PAE, enzymes, and genes involved in P recycling under P deficiency resulted in a higher leaf and root biomass compared to that of both untreated plants and those treated with H2. Moreover, distinct contents of endogenous phytohormones and phenols in humates likely accounted for their different capacities to stimulate plant growth. H1 also enhanced the antioxidant capacity in leaves and roots more than H2 did, contributing to plant resilience against abiotic stress, including P limitation. Overall, this study underscores the potential of H1 as an efficient biostimulant, especially in P-limited conditions, through mechanisms involving hormonal modulation, antioxidant reinforcement, and improved P uptake and recycling.

## Figures and Tables

**Table 1:** Chemical, spectroscopic and thermal parameters of humates H1 and H2. R1, R2, R3: ratios between integrated areas calculated from ^13^C NMR spectra; WL _x/y_: ratio between weight losses (WL) occurring within different temperature ranges; TG-T_50_: temperature at which half of biomass is lost; IAA: indoleacetic acid; ABA: abscisic acid; GA: gibberellic acid; TP: total phenols; FRAP = ferric-reducing antioxidant power assay. Values are reported as the average of 5 technical replicates (±SD). For each parameter, different lowercase letters indicate significant differences between humates at *p* < 0.05.

Parameter	H1	H2
N (%)	1.64 ± 0.03 a	1.49 ± 0.04 b
C (%)	42.02 ± 0.07 a	39.52 ± 0.10 b
H (%)	5.36 ± 0.07 a	4.96 ± 0.11 b
S (%)	0.07 + 0.00 b	0.14 + 0.01 a
O (%)	50.97 + 0.07 b	54.01 + 0.09 a
C/N	25.66 + 0.37 a	26.50 + 0.66 a
C/H	7.84 + 0.12 a	7.97 + 0.19 a
C/O	0.82 + 0.00 a	0.73 + 0.00 b
Ash (%)	28.8	30.8
R1	1.8	1.1
R2	0.25	0.36
R3	0.25	0.20
WL_400–550/200–300_	0.459	0.567
WL_400–550/300–400_	0.916	0.947
TG-T_50_ (°C)	364	407
IAA (ng mg C^−1^)	0.13 ± 0.06 a	0.13 ± 0.04 a
Zeatin riboside (ng mg C^−1^)	1.86 ± 0.16 a	1.56 ± 0.19 b
ABA (ng mg C^−1^)	9.59 ± 2.36 a	6.99 ± 1.93 a
GA (ng mg C^−1^)	0.013 ± 0.004 a	0.104 ± 0.015 b
Acidity (meq H^+^ mg^−1^)	2.50 ± 0.05 b	3.33 ± 0.09 a
TP (mg GAE kg^−1^)	89.31 ± 0.16 a	59.43 ± 0.07 b
FRAP (mg Fe^2+^ kg^−1^)	536.5 + 0.10 a	72.11 + 0.01 b

**Table 2:** Carbon (C) and nitrogen (N) content (mean ± SD) in leaves and roots of maize plants treated with humates H1 and H2, and of untreated plants (control). Plants were supplied with low (25 μM)- or high (250 μM)-P concentration. Different lowercase letters indicate significant differences between control and humate-treated plants along each column within a specific P group (*p* < 0.05).

	Leaves		Roots	
	*N* (*%*)	*C* (*%*)	*N* (*%*)	*C* (*%*)
	Low P			
**Control**	4.75 ± 0.47 b	34.00 ± 1.51 a	3.98 ± 0.31 b	31.51 ± 1.58 a
**H1**	5.27 ± 0.34 a	33.35 ± 1.48 a	4.61 ± 0.10 a	31.87 ± 1.19 a
**H2**	5.11 ± 0.26 a	32.94 ± 1.26 a	4.71 ± 0.20 a	32.11 ± 0.57 a
		**High P**		
**Control**	5.00 ± 0.01 b	33.82 ± 0.76 a	4.93 ± 0.32 b	28.91 ± 1.80 c
**H1**	5.72 ± 0.36 a	34.50 ± 0.78 a	5.35 ± 0.39 ab	32.21 ± 0.38 b
**H2**	5.52 ± 0.17 a	33.10 ± 3.11 a	5.69 ± 0.40 a	34.41 ± 0.08 a

**Table 3:** Ferric-reducing antioxidant power (FRAP) in leaves and roots (mean ± SD). F.W. = fresh weight. Different lowercase letters indicate significant differences between control and humate-treated plants along each column within a specific P group (low or high) (*p* < 0.05).

	Leaves	Roots	Leaves	Roots
	mg Fe^2+^ kg^−1^ F.W.		mg Fe^2+^ kg^−1^ F.W.	
	Low P		High P	
**Control**	0.41 ± 0.05 b	0.51 ± 0.13 b	0.51 ± 0.13 b	0.26 ± 0.05 b
**H1**	0.64 ± 0.05 a	0.75 ± 0.03 a	0.75 ± 0.03 a	0.41 ± 0.04 a
**H2**	0.52 ± 0.04 a	0.63 ± 0.05 b	0.63 ± 0.05 b	0.33 ± 0.06 ab

**Table 4:** Number of differentially expressed genes (DEGs) in the different comparisons.

	Number of DEGs	
Comparisons	Overexpressed	Underexpressed
HP vs. LP	528	239
HP vs. HP_H1	245	278
LP vs. LP_H1	179	164

**Table 5:** Selected differentially expressed genes (DEG) for the three comparisons considered.

Gene ID	Log2FC	*p*-Value	Description
**DEG in HP–LP**			
*Zm00001d049554*	−2.4766	0.0001	Putative glycerol-3-phosphate transporter 1
*Zm00001d026156*	−1.6923	0.0007	glycerol 3-phosphate permease
*Zm00001d008310*	−1.8869	0.0176	inositol-1-monophosphatase
*Zm00001d043267*	−1.3904	0.0325	putative 1-acyl-sn-glycerol-3-phosphate acyltransferase 4
*Zm00001d011734*	−1.9039	0.0406	phosphatase phospho 1
*Zm00001d031653*	−1.6914	1.9 × 10^−5^	uncharacterized LOC100216744
*Zm00001d040519*	−2.1226	0.015557	rhicadhesin receptor
*Zm00001d049958*	−1.6753	0.009485	Putative peptidyl-prolyl cis-trans isomerase WD40 repeat domain family protein
*Zm00001d053952*	−1.4504	0.038968	Bax inhibitor 1
*Zm00001d039439*	−1.517	0.0272	Protein BRASSINAZOLE-RESISTANT 1
*Zm00001d046538*	−1.1071	0.024709	SEC14-like protein 1
*Zm00001d013869*	−1.2837	0.03877	SAUR56-auxin-responsive SAUR family member
*Zm00001d028370*	−1.1231	0.017965	protein OBERON 3
**DEG in HP-HP_H1**			
*Zm00001d014564*	−1.3433	0.042067	sulfate transporter 6
*Zm00001d012913*	−1.0042	0.025655	methionine adenosyltransferase
*Zm00001d039138*	−1.1375	0.016453	methionine aminopeptidase
*Zm00001d012537*	−1.223	0.029047	somatic embryogenesis receptor-like kinase 3
*Zm00001d003306*	−1.7652	0.003905	putative LRR receptor-like serine/threonine-protein kinase
*Zm00001d053967*	−1.0378	0.045439	auxin response factor 21
*Zm00001d038508*	−1.3427	0.014187	auxin response factor
*Zm00001d008893*	−1.7031	0.005967	auxin response factor 10
*Zm00001d021526*	−1.2159	0.017747	SNARE-interacting protein KEULE
*Zm00001d032380*	−1.0691	0.041987	SCARECROW-like protein
*Zm00001d050810*	−1.5393	0.007842	WAT1-related protein
*Zm00001d024854*	−1.2124	0.015484	trehalose-6-phosphate synthase
*Zm00001d014811*	−1.3976	0.000873	alkaline alpha galactosidase 3
*Zm00001d042025*	−1.525	0.00095	probable galactinol--sucrose galactosyltransferase 1
*Zm00001d039029*	−1.0826	0.033409	60 kDa jasmonate-induced protein
*Zm00001d018797*	−1.4042	0.034359	putative glycogen synthase kinase family protein
*Zm00001d044644*	−1.6404	0.020783	Anamorsin homolog
*Zm00001d031941*	−1.6483	6 × 10^−5^	chaperone DNA J2
*Zm00001d011454*	−2.734	0.000183	Zinc finger CCCH domain-containing protein 19
*Zm00001d013202*	−9.4918	0.03132	DNA-directed RNA polymerase subunit beta’-like
*Zm00001d014937*	−2.1111	0.005352	CBF1-interacting co-repressor CIR N-terminal
*Zm00001d003306*	−1.7652	0.003905	LRR receptor-like serine/threonine-protein kinase
**DEG in LP-LP_H1**			
*Zm00001d039644*	−5.3403	0.005928	cytokinin-O-glucosyltransferase 3
*Zm00001d040011*	−3.5851	0.045957	Protein ABA DEFICIENT 4 chloroplastic
*Zm00001d016132*	−4.3379	0.016201	AT-hook motif nuclear-localized protein 26
*Zm00001d013655*	−4.1118	0.021104	nicotianamine synthase 3
*Zm00001d017501*	−3.8926	0.030827	peroxisomal biogenesis factor 19
*Zm00001d025296*	−3.43	0.014938	nudix hydrolase 13
*Zm00001d052702*	−3.4285	0.012467	biotin synthase
*Zm00001d003522*	−3.1687	0.000711	Pyridoxal phosphate (PLP)-dependent transferase superfamily protein
*Zm00001d012508*	−3.1634	0.017243	WRKY22-superfamily of TFs having WRKY and zinc finger domains
*Zm00001d032692*	−1.7173	0.047759	MATH domain containing protein
*Zm00001d032576*	−2.7589	0.009631	protochlorophyllide reductase B
*Zm00001d049886*	−2.6671	0.039014	grx_S12-glutaredoxin subgroup I
*Zm00001d044606*	−1.3068	0.033098	grx_S16-glutaredoxin subgroup II
*Zm00001d037993*	−2.6568	0.006811	ran-binding protein 1
*Zm00001d029457*	−2.4776	0.006301	nucleoredoxin 1
*Zm00001d029361*	−2.3294	0.020461	zinc finger CCCH domain-containing protein 15 homolog
*Zm00001d047802*	−1.553	0.045089	Dof zinc finger protein DOF2.2
*Zm00001d037221*	−1.2931	0.014043	Transcription factor TCP14
*Zm00001d050107*	−2.1979	0.017464	12-oxophytodienoate reductase 8
*Zm00001d047105*	−3.037	0.035362	Oxysterol-binding protein-related protein 2A
*Zm00001d045298*	−1.4759	0.024132	Lipase-like
*Zm00001d033905*	−2.0678	0.041084	growth-regulating-factor-interacting factor 1
*Zm00001d034035*	−1.4166	0.031865	oligopeptide transporter 3

## Data Availability

Not applicable.

## Supplementary Materials

The following supporting information can be downloaded at https://www.mdpi.com/article/10.3390/plants12183291/s1. Table S1: Complete list of DEGs and GO analysis. Figure S1: ATR FT-IR spectra of lyophilized leaves of maize plants grown at low P (A) or high P (B) concentration. The red line corresponds to the control, while the black or bleu lines to H1.
