# Supplementary figures and images for: Phosphorus Acquisition Efficiency and Transcriptomic Changes in Maize Plants Treated with Two Lignohumates
Source: Plants (Basel). 2023 Sep 17;12(18):3291. doi: 10.3390/plants12183291 (PMC10535022; doi:10.3390/plants12183291)

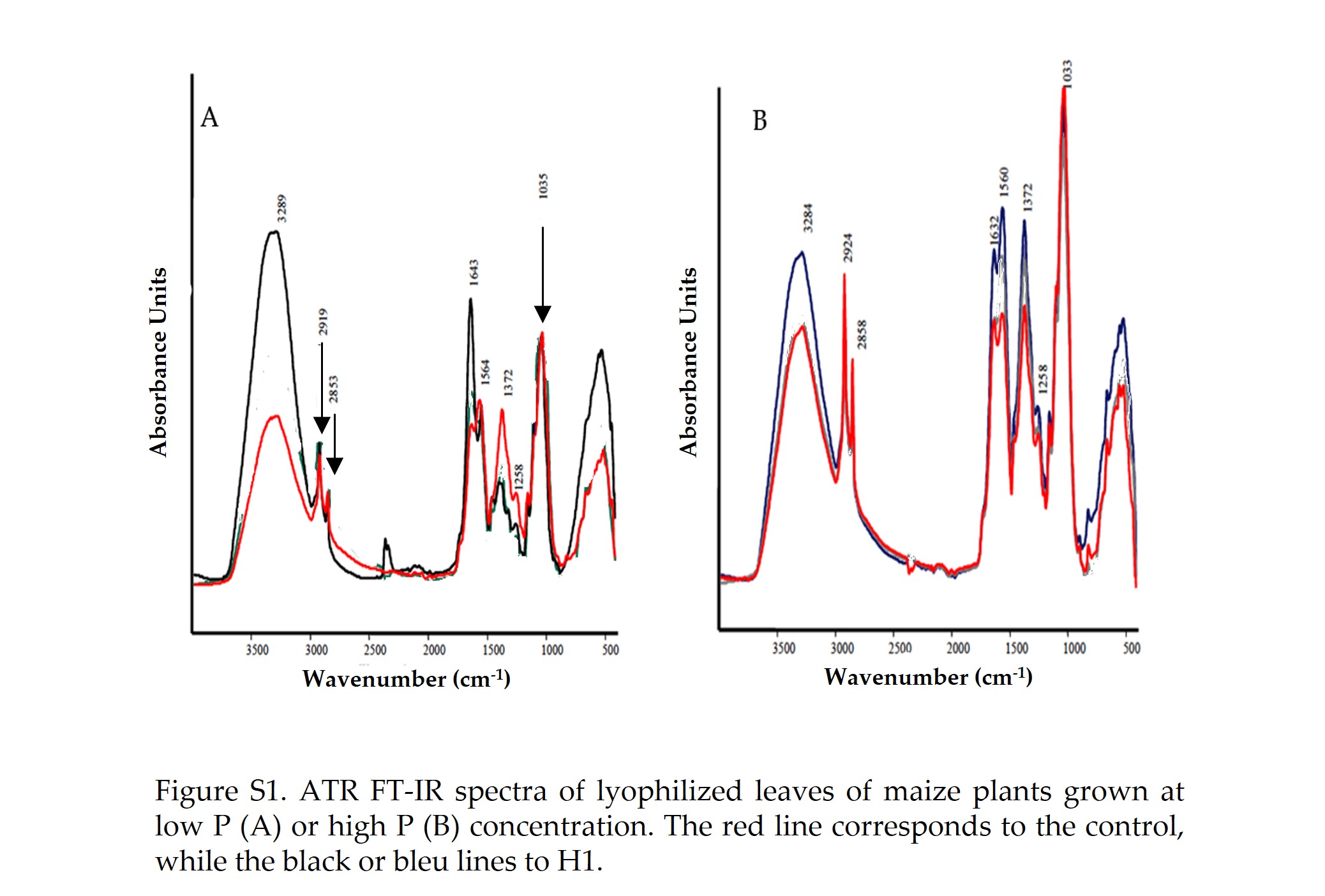

Supplement: Supplementary file 1 [file plants-12-03291-s001.zip › Supplementary Figure S1.jpg]
